# Five new species of the armored scale genus *Andaspis* MacGillivray (Hemiptera, Coccomorpha, Diaspididae) from New Caledonia

**DOI:** 10.3897/zookeys.693.13074

**Published:** 2017-08-22

**Authors:** Fredericka B. Hamilton, Douglas J. Williams, Nate B. Hardy

**Affiliations:** 1 Auburn University, Department of Entomology and Plant Pathology, 301 Funchess Hall, Auburn, AL 36849, USA; 2 The Natural History Museum, Department of Life Sciences (Entomology), Cromwell Road, London SW7 5BD, UK

**Keywords:** Alpha-taxonomy, armored scale insects, Southern Hemisphere biota

## Abstract

New Caledonia is home to many endemic species of plants and animals. Here, we improve our grasp on that biota by describing five new species of armored scale insects in the genus *Andaspis*: *Andaspis brevicornuta* **sp. n**, *A. conica* **sp. n.**, *A. nothofagi* **sp. n.**, *A. novaecaledoniae* **sp. n.**, and *A. ornata* **sp. n.** Each is known exclusively from collections on southern beeches (*Nothofagus* spp.) in New Caledonia. A key to the species of *Andaspis* of New Caledonia is provided.

## Introduction

New Caledonia is an archipelago located in the South Pacific Ocean east of Australia. It was once part of the ancient supercontinent Gondwana, but has been isolated for approximately 80 million years (Grandcolas et al. 2008). It encompasses an array of soil types and biomes – including evergreen humid forests, sclerophyll dry forests, maquis, and savannas (Murienne 2009). Due to its isolation and geographic heterogeneity, much of New Caledonian biodiversity is endemic and New Caledonia is considered to be a biodiversity hotspot (Ladiges and Cantrill 2007, Grandcolas et al. 2008). Among the emblematic elements of the New Caledonian biota are the southern beeches (*Nothofagus* spp.) (Nothofagaceae). Southern beeches are archetypal Gondwanan plants, which might suggest biotic interchanges with other Southern Hemisphere landmasses when they were contiguous (Cook and Crisp 2005, Ladiges and Cantrill 2007). However, the ultramafic soils which cover much of the surface of New Caledonia suggest a period in which it was entirely submerged (Grandcolas et al. 2008). Hence, although southern beeches may have long been an important component of the New Caledonian terrestrial biota, the ones found there today must have dispersed over an ocean, from elsewhere in the Southern Hemisphere. Here, we improve our characterization of New Caledonian biota by describing five new species of armored scale insects associated with *Nothofagus*.

An estimated 4,000 species of insects have been recorded from New Caledonia (Lowry et al. 2004) and 118 of these are scale insects (Hemiptera: Coccomorpha) (Mille et al. 2016). Scale insects mostly feed on plant sap or the contents of plant cells. They live on a variety of host-plant species, and many are ecological and agricultural pests (Campbell et al. 2014). The New Caledonian species represent ten scale insect families: Asterolecaniidae, Coccidae, Conchaspididae, Dactylopiidae, Diaspididae, Eriococcidae, Monophlebidae, Ortheziidae, Pseudococcidae, and Rhizoecidae (Mille et al. 2016).

The most species-rich family of scale insects is Diaspididae, colloquially referred to as armored scale insects. We currently recognize 2,650 valid species of armored scale insects, in 418 genera (García Morales et al. 2016). Fifty species of armored scale insects and 26 genera have been collected from New Caledonia. About 20% are thought to be endemic (Mille et al. 2016). Given its long history of isolation, this may seem to be a strikingly low proportion. It can be explained in part by the fact that scale insects are extremely invasive (Miller et al. 2005). But the low proportion of endemic species could also reflect that the scale insect fauna has been little studied; much of the endemic diversity could be undocumented. In what follows, we describe five new species of New Caledonian endemic armored scales in the genus *Andaspis* MacGillivray, a genus which had not yet been recorded from New Caledonia. Before this work, *Andaspis* comprised 46 species distributed mostly throughout Eastern Asia and Australia (Northern Territory and Queensland) (Williams and Watson 1988, García Morales et al. 2016).

With the additions made here, the total number of described species in *Andaspis* is 51. We provide a key to the New Caledonian species.

## Materials and methods

The following descriptions are based on forty-six slide-mounted specimens of adult female scale insects which had been prepared from material collected by J. S. Dugdale and P. N. Johnson in 1978. All of the specimens examined were collected from two sites in New Caledonia: Mont Mou and Rivière Bleue. These specimens are part of the scale insect collection of The Natural History Museum, London, UK (BMNH). D. J. Williams delimited the species, and F. B. Hamilton described them. They are joint authors of the five new species names introduced below.

Specimens were viewed through a phase contrast light microscope. NIS elements software was used to take photographs and measurements. All of the given measurements are maximum dimensions and are expressed as a range across specimens. The body of the adult female and a higher magnification image of the pygidium were traced and inked from the photographs. Illustrations were refined with the Adobe Creative Suite. In each of the illustrations, the dorsal body surface is displayed on the left side and the ventral body surface on the right side. Surrounding the main image are enlargements of diagnostic features. Measurements are given in millimeters (mm) and micrometers (µm). The morphological terminology used in the descriptions follows that of Williams and Watson (1988) and Miller and Davidson (2005). Specimens are deposited at BMNH and NMNH (National Museum of Natural History, Beltsville, MD).

## Taxonomy

### Andaspis

Taxon classificationAnimaliaHemipteraDiaspididae

MacGillivray, 1921

Andaspis MacGillivray, 1921: 275; Rao and Ferris 1952: 17; Williams 1963: 13; Takagi 1970: 20; Williams and Watson 1988: 27.

#### Type species.

Mytilaspis flava var. hawaiiensis Maskell, 1895, by original designation

#### Generic diagnosis.

Body shape of adult female variable: oblong, fusiform, obovate, or oval. Antennae with 2 or more setae. Prominent median lobes with short inner edges, long outer margins, and conspicuous scleroses and paraphyses. A pair of small gland spines between lobes. Second lobes greatly reduced or absent and 4–6 marginal macroducts present on each side of pygidium. Anal opening in anterior portion of pygidium or near apex. Vulva either without perivulvar pores or surrounded by three or five groups of them (Takagi and Kawai 1966, Takagi 1970, Williams and Watson 1988).

### Andaspis brevicornuta

Taxon classificationAnimaliaHemipteraDiaspididae

Hamilton & Williams sp. n.

http://zoobank.org/4D4A2A7C-9239-4054-9F31-18EC93C1ADED

Figures 1–6

#### Material examined.

Holotype: adult female, slide-mounted. Original label: “New Caledonia, Rivière Bleue, *Nothofagus codonandra*, J.S. Dugdale, 10.x.1978, *Andaspis*” (handwritten in black ink). Deposited at BMNH.

#### Description.

**Adult female.** Slide-mounted adult female 1.06 mm long; widest at third abdominal segment, 0.48 mm wide. Body outline oblong, derm membranous except for pygidium. A distinctive species with six spurs or minute horns present on anterior edge of head. Each antenna with five setae. Anterior spiracles each with 2 disc pores, each about 4 µm in diameter, trilocular; posterior spiracles lacking pores. Anterior abdominal segments well-developed with convex margins; tooth-like tubercles present on segments 2 and 4. In addition to those on pygidium, gland spines located along margins of abdominal segments 3 and 4. Many short macroducts distributed around ventral margins extending from mesothorax to abdominal segment 1.

Pygidium with well-developed median lobes, approximately triangular in shape. Two short gland spines present between median lobes. Each median lobe with paraphysis arising from outer and inner basal angles, anterior ends almost touching, also with short sclerosis arising from inner angle and a large club-shaped sclerosis arising from outer basal angle. Second lobes present; each with a large club-shaped basal sclerosis. Third lobes present, each with outer edge notched but without basal sclerosis. Additionally, a pointed tubercle located on each side of abdominal segment 6. Eight gland spines present along the margin of each side of the pygidium, each with a long microduct, about 40 µm. Marginal setae present, each about 14 µm long, setae on abdominal segment 7 shorter, about 10 µm long. Macroducts on pygidium restricted to dorsal margin and submargin, with eight marginal macroducts and five smaller and narrower submarginal macroducts located on each side. Marginal macroduct openings narrowly oval, each about 8 µm long × 3 µm wide. Opening of each macroduct on segment 7 narrow, 5 µm long × 3 µm wide. Perivulvar pores absent. Identity of dark-rimmed circular structures on venter and dorsum of pygidium near vulva unknown and they could be orifices of pores or setal sockets.

**Figure 1–6.**
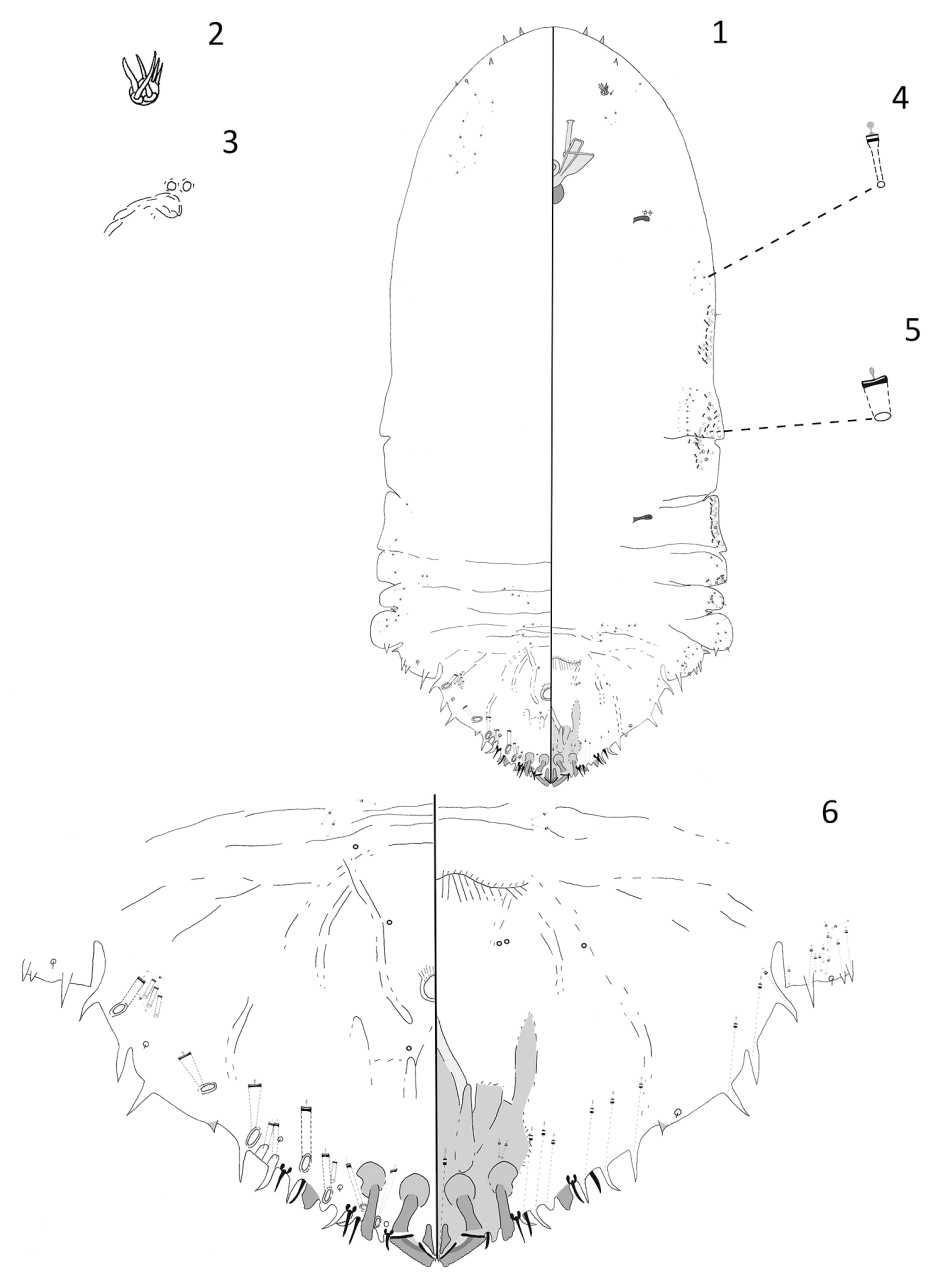
*Andaspis brevicornuta* Hamilton and Williams, sp. n., adult female; **1** whole body **2** antenna **3** anterior spiracle **4** microduct **5** macroduct **6** pygidium.

#### Remarks.

The adult female of this species differs from those of all other currently described *Andaspis* species by having 6 tooth-like spurs (or minute horns) present on the anterior margin of the head. This species is somewhat similar to *Andaspis halli* Rao, 1952, a species known to occur in Zimbabwe. Adult females of the two species share an elongate body shape and a distinct second lobe. However, this species differs from it by the following characters (those for *A. halli* in parentheses): two scleroses located above each median lobe (no scleroses present above each median lobe), a club-shaped sclerosis present above each second lobe (an elongate sclerosis present above each second lobe), eight marginal macroducts located on the dorsum (six marginal macroducts located on the dorsum), and lacking perivulvar pores (three groups of perivulvar pores).

#### Etymology.

The specific epithet is the Latin adjective meaning short horned, referring to the projections on the head margin.

### Andaspis conica

Taxon classificationAnimaliaHemipteraDiaspididae

Hamilton & Williams sp. n.

http://zoobank.org/F31D9577-607E-4E92-9F0E-E24418422622

Figures 7–11

#### Material examined.

Holotype: adult female, slide-mounted. Original label: “New Caledonia, Rivière Bleue, *Nothofagus codonandra*, J.S. Dugdale, 10.x.1978, *Andaspis*” (handwritten in black ink). Deposited at BMNH.

Paratypes: 13 adult females. Same data as holotype. Deposited at BMNH and NMNH.

#### Description.

**Adult female.** Slide-mounted adult female 0.88–1.52 mm long; 0.42–0.54 mm wide. Body outline fusiform, derm membranous except for pygidium. Each antenna with four setae. Anterior spiracles each with 1 or 2 disc pores, each about 3 µm in diameter, trilocular; posterior spiracles lacking pores. Anterior abdominal segments well-developed with convex margins; tooth-like tubercles present on margins of segments 1, 3, and 4. In addition to those on pygidium, a pair of gland spines present along lateral margins of abdominal segment 4.

Pygidium with well-developed median lobes, approximately triangular in shape. Two short gland spines present between median lobes, extending almost halfway down lobes. Each median lobe with a transversal paraphysis arising from each basal angle, inner ends almost touching; a short sclerosis arising from inner basal angle, and a longer club-like sclerosis extending from lateral basal angle of each median lobe. Second lobes present; each with a short pyriform sclerosis near base. Eleven gland spines present on each lateral margin of pygidium, each gland spine with a long microduct, about 45 µm. Marginal setae each about 12 µm in length, setae on abdominal segment 7 shorter, each about 9 µm long. Macroducts on pygidium restricted to margin and submargin. Four marginal macroducts and one smaller, narrower submarginal macroduct located on each side of dorsum. Macroduct openings almost vertical to margin, narrowly oval, each about 11 µm long × 3 µm wide, except for a very narrow submarginal macroduct located on abdominal segment 7 with an opening 9 µm long × 2 µm wide. Perivulvar pores absent. Identity of dark-rimmed circular structures on venter and dorsum of pygidium near vulva unknown and they could be orifices of pores or setal sockets.

**Figure 7–11.**
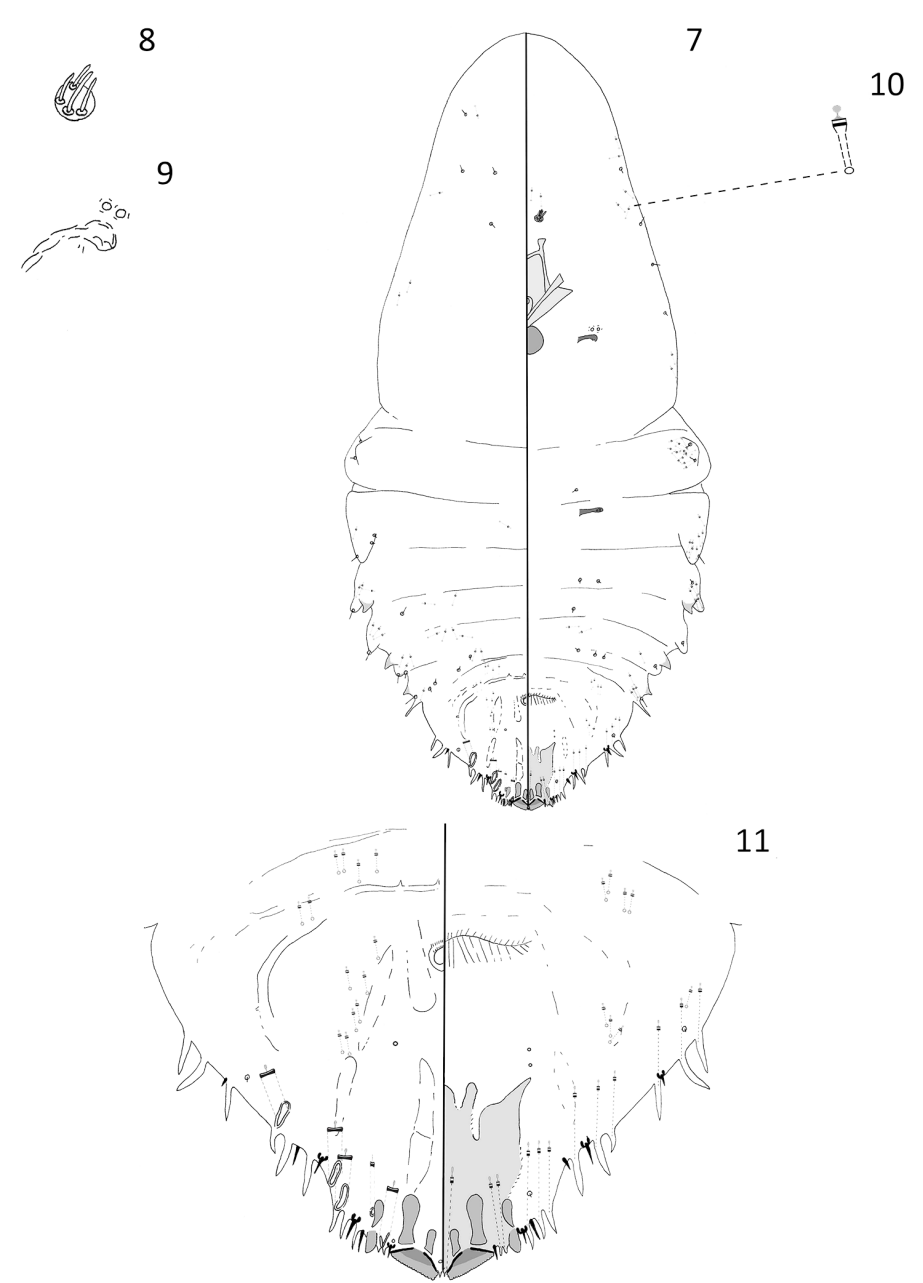
*Andaspis conica* Hamilton and Williams, sp. n., adult female; **7** whole body **8** antenna **9** anterior spiracle **10** microduct **11** pygidium.

#### Remarks.

The adult female of this species most resembles that of *Andaspis kazimiae* Williams, 1963, a species known to occur in Pakistan. Adult females of the two species share four marginal macroducts located on the dorsum and have a second lobe. This species differs from *A. kazimiae* by the following characters (those for *A. kazimiae* in parentheses): a pair of scleroses located above each median lobe (scleroses absent above each median lobe), second lobe with pyriform sclerosis near base (second lobe without pyriform sclerosis near base), lacking perivulvar pores (three groups of perivulvar pores), and antennae with four setae (antennae with two setae).

#### Etymology.

The specific epithet *conica* is the Latin feminine adjective meaning conical and refers to the conical head.

### Andaspis nothofagi

Taxon classificationAnimaliaHemipteraDiaspididae

Hamilton & Williams sp. n.

http://zoobank.org/0073B165-8A89-4163-B2CF-14B3D2DA7629

Figures 12–16

#### Material examined.

Holotype: adult female, slide-mounted. Original label: “New Caledonia, Mt. Mou, *Nothofagus baumanii* twigs, P.N. Johnson, 2.xi.1978, *Andaspis*” (handwritten in black ink). Deposited at BMNH.

Paratypes: 5 adult females. Same data as holotype. Deposited at BMNH and NMNH.

#### Description.

**Adult female.** Slide-mounted adult female 1.15–1.72 mm long; widest at first abdominal segment, 0.64–0.74 mm wide. Body outline oval or oblong, derm membranous except for pygidium. Each antenna with three setae. Anterior spiracles each with 3–5 disc pores, each about 4 µm in diameter, trilocular; posterior spiracles lacking pores. Anterior abdominal segments well-developed with convex margins; tooth-like tubercles present on margins of segments 1, 3, and 4. In addition to those on pygidium, a pair of gland spines along margin of abdominal segment 4 on each side. Many microducts distributed along margins of abdomen, thorax, and head.

Pygidium with well-developed median lobes, approximately triangular in shape. Two short gland spines present between lobes. Each median lobe with a paraphysis extending anterolaterally from inner angle, and another extending medially from outer angle with inner ends almost touching. A short sclerosis arising from inner base of each median lobe and a longer club-like sclerosis arising from near lateral base. Second and third lobes present; second lobes each with an hourglass-shaped sclerosis arising from base and third lobes each with a short oval sclerotic base. Seven to eight gland spines present along margin of each side of pygidium, each with long microducts, each about 60 µm. Marginal setae each about 15 µm in length, setae on abdominal segment 7 shorter, about 11 µm long. Macroducts on pygidium restricted to margin and submargin. Four marginal macroducts and one smaller and narrower submarginal macroduct located on each side of dorsum. Macroduct openings narrowly oval, each about 9 µm long × 4 µm wide. Macroduct located on abdominal segment 7 with a much narrower opening compared to the others, about 8 µm long × 2 µm wide. Perivulvar pores absent. Identity of dark-rimmed circular structures on dorsum and venter of pygidium near vulva unknown and they could be orifices of pores or setal sockets.

**Figure 12–16.**
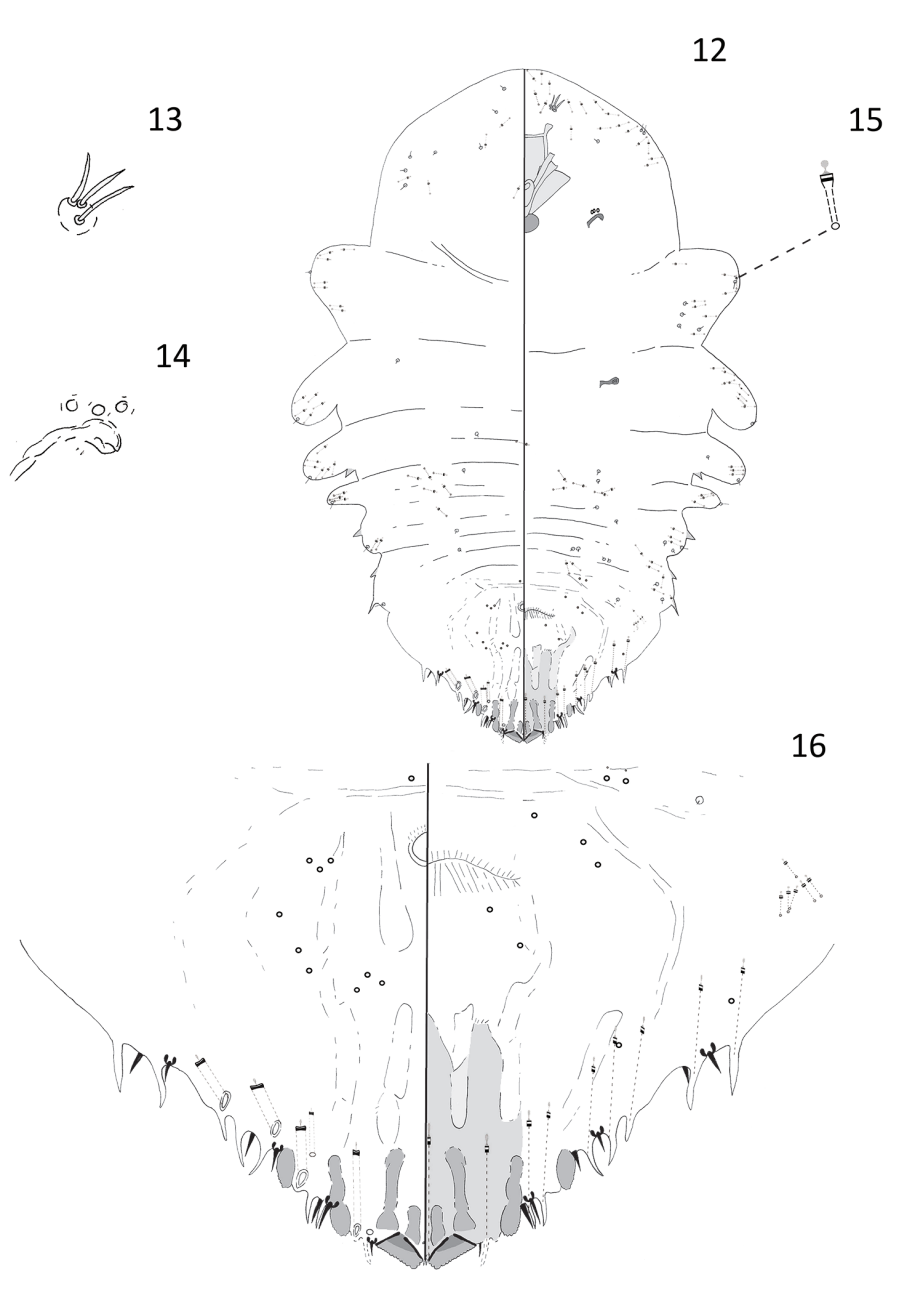
*Andaspis nothofagi* Hamilton and Williams, sp. n., adult female; **12** whole body **13** antenna **14** anterior spiracle **15** microduct **16** pygidium.

#### Remarks.

The adult female of this species is somewhat similar to that of *Andaspis laingi* Rao, 1952, a species known to occur in India. Adult females of the two species share an oval body shape and four marginal macroducts located on the dorsum. This species differs from *A. laingi* by the following characters (those for *A. laingi* in parentheses): well-developed lateral lobes (slightly developed lateral lobes), two scleroses present above each median lobe (one paraphysis above each lobe), and, in addition a short sclerosis present above each second lobe (sclerosis absent and second lobe obsolete), and a short sclerotic area present above each third lobe (sclerotic area absent and third lobe obsolete), also lacking perivulvar pores (three groups of perivulvar pores).

#### Etymology.

The specific epithet is the Latin genitive of the host plant genus, *Nothofagus*.

### Andaspis novaecaledoniae

Taxon classificationAnimaliaHemipteraDiaspididae

Hamilton & Williams sp. n.

http://zoobank.org/5368CA1F-D60B-49FC-A36B-4BCB2BC8144C

Figures 17–22

#### Material examined.

Holotype: adult female, slide-mounted. Original label: “New Caledonia, Rivière Bleue, *Nothofagus codonandra*, J.S. Dugdale, 10.x.1978, *Andaspis*” (handwritten in black ink). Deposited at BMNH.

Paratypes: 21 adult females. New Caledonia: Rivière Bleue and Mt. Mou. Collected on *Nothofagus baumanii* and *N. codonandra*, J.S. Dugdale and P.N. Johnson, 10.x.1978 and 2.xi.1978. Deposited at BMNH and NMNH.

#### Description.

**Adult female.** Slide-mounted adult female 0.84–1.46 mm long; widest at first abdominal segment, 0.52–0.84 mm. Body outline oval or oblong, derm membranous except for pygidium. Each antenna with three setae. Anterior spiracles each with 1–4 disc pores, each about 5 µm in diameter, trilocular; posterior spiracles lacking pores. Anterior abdominal segments well-developed with convex margins; tooth-like tubercles present on segments 1, 3, and 4. In addition to those on pygidium, gland spines present along margins of abdominal segments 3 and 4. Many microducts distributed along margins and submargins of thorax and abdomen on both venter and dorsum, plus several on head.

Pygidium with well-developed median lobes, each approximately triangular in shape. Two short gland spines present between median lobes, extending almost halfway down lobes. Each median lobe with a paraphysis arising from outer angle of lobe and another arising from inner basal angle of lobe, both paraphyses often pointing medially with inner ends almost touching. A short sclerosis arising from inner basal part of lobe and a longer club-like sclerosis extending from lateral half. Second lobes present; short and pointed, each with a short sclerosis arising from base. Eight gland spines present along margin of each side of pygidium, each with a long microduct, about 90 µm in length. Marginal setae each about 16 µm long, setae on abdominal segment 7 shorter, about 13 µm long. Macroducts on pygidium restricted to margin and submargin. Five marginal macroducts located on each side of dorsum and two on venter. Macroduct openings narrowly oval, almost perpendicular to margin, each about 13 µm long × 3 µm wide. Each macroduct located on segment 7 with much narrower opening compared to others, about 8 µm long × 2 µm wide. Two narrower submarginal macroducts located posterior to macroducts on dorsum on segments 6 and 7, each opening about 6 µm long × 3 µm wide. Perivulvar pores absent. Identity of dark-rimmed circular structures on venter and dorsum of pygidium near vulva unknown and they could be orifices of pores or setal sockets.

**Figure 17–22.**
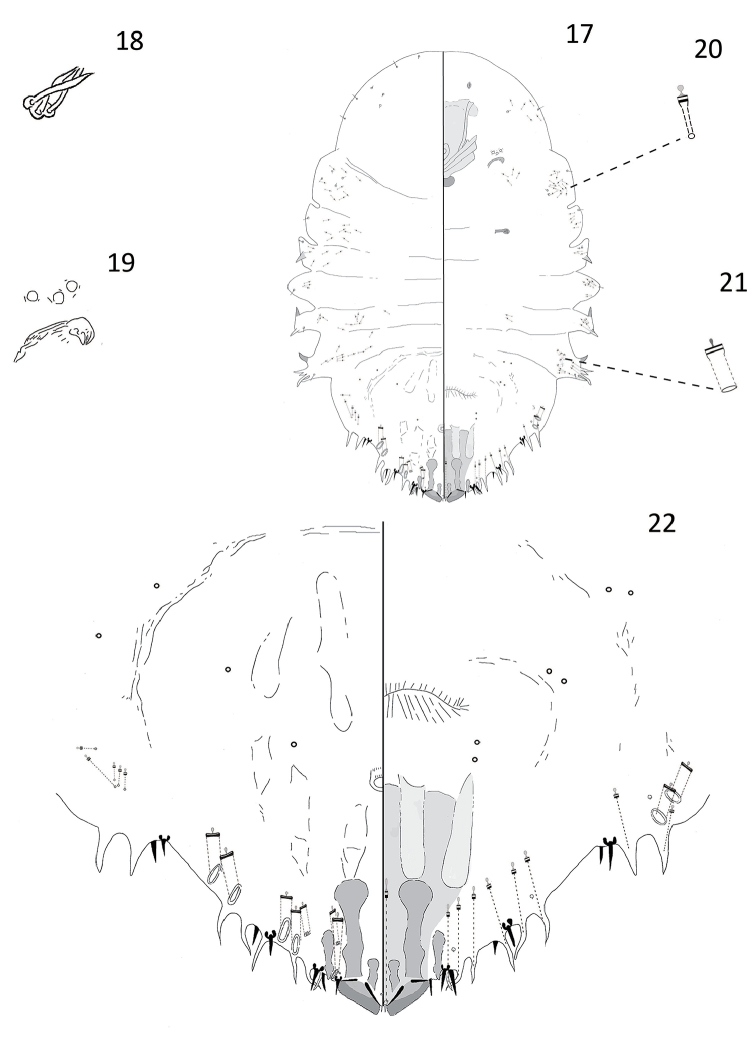
*Andaspis novaecaledoniae* Hamilton and Williams, sp. n., adult female; **17** whole body **18** antenna **19** anterior spiracle **20** microduct **21** macroduct **22** pygidium.

#### Remarks.

The adult female of this species is different from those of all other species in the genus described so far, in having two marginal macroducts located on the venter. Similarly, *A. ornata* sp. n. has nine marginal macroducts located on the venter. However, this species is somewhat similar to *Andaspis tokyoensis* Takagi and Kawai, 1966, a species known to occur in Japan. Adult females of *A. novaecaledoniae* and *A. tokyoensis* share well-developed lateral lobes on the abdomen, a club-shaped sclerosis arising from each median lobe, a narrow macroduct located on abdominal segment 7, and a sclerosis located anterolateral to each median lobe. This species differs from *A. tokyoensis* by the following characters (those for *A. tokyoensis* in parentheses): two scleroses located above each median lobe (one sclerosis located above each median lobe), five marginal macroducts and two submarginal macroducts located on the dorsum (six marginal macroducts and one submarginal macroduct located on the dorsum), lacking perivulvar pores (three groups of perivulvar pores), and antennae with three setae (antennae with two setae).

#### Etymology.

The specific epithet is taken from the latinized name of the country in which it occurs meaning “of New Caledonia”.

### Andaspis ornata

Taxon classificationAnimaliaHemipteraDiaspididae

Hamilton & Williams sp. n.

http://zoobank.org/F90EFDEA-5B2C-437E-8419-065C50C6AF65

Figures 23–29

#### Material examined.

Holotype: adult female, slide-mounted. Original label: “New Caledonia, Mt. Mou, *Nothofagus baumanii* twigs, P.N. Johnson, 2.xi.1978, *Andaspis*” (handwritten in black ink). Deposited at BMNH.

Paratypes: 2 adult females. Same data as holotype. Deposited at BMNH and NMNH.

#### Description.

**Adult female.** Slide-mounted adult female 1.36–2.72 mm long; widest at mesothorax, 0.90–1.67 mm. Body outline obovate, derm membranous except for pygidium. Each antenna with four setae. Anterior spiracles each with 1–2 disc pores, each about 4 µm in diameter, indistinguishable number of loculi; posterior spiracles lacking pores. A prominent cicatrix located on each side of mesothorax on dorsal side of body. Anterior abdominal segments well-developed with convex margins; tooth-like tubercles present on margins of segments 1, 3, and 4. In addition to gland spines on pygidium, gland spines also present along the margins of abdominal segments 3 and 4. Many microducts located on the dorsum of the metathorax and abdominal segments 1, 2, 3, and 4. Short macroducts present along margins of venter.

Pygidium considerably shorter and narrower in comparison to rest of body, with well-developed median lobes that are approximately triangular in shape. Two short gland spines present between median lobes. Each median lobe with a paraphysis arising from inner and outer basal angles, ends almost touching. Each median lobe with a short medial sclerosis arising from inner basal angle and a longer club-like sclerosis extending from lateral basal angle. Second lobes present, rounded, much smaller than median lobes, each with a short basal sclerosis. Third lobes short and rounded, each with a short sclerotized area extending along margin. Eight gland spines present along the margin of each side of the pygidium, each gland spine with a long microduct, about 35 µm. Marginal setae on pygidium each about 20 µm in length, setae on abdominal segment 7 shorter, about 15 µm long. Macroducts on pygidium restricted to margin and submargin. Five marginal macroducts and one smaller and narrower submarginal macroduct located on each side of the dorsum; macroduct openings on dorsum narrowly oval, each about 8 µm long × 4 µm wide. Nine marginal macroducts located on each side of the venter; openings of marginal macroducts on venter slightly larger and nearly circular in shape, about 9 µm long × 7 µm wide. Anal opening in the adult female of this species is placed close to pygidium apex. Apex of pygidium to anal opening about 53 µm; apex of pygidium to vulva about 213 µm. Perivulvar pores absent. Identity of dark-rimmed circular structures on venter and dorsum of pygidium near vulva unknown and they could be orifices of pores or setal sockets.

**Figure 23–29.**
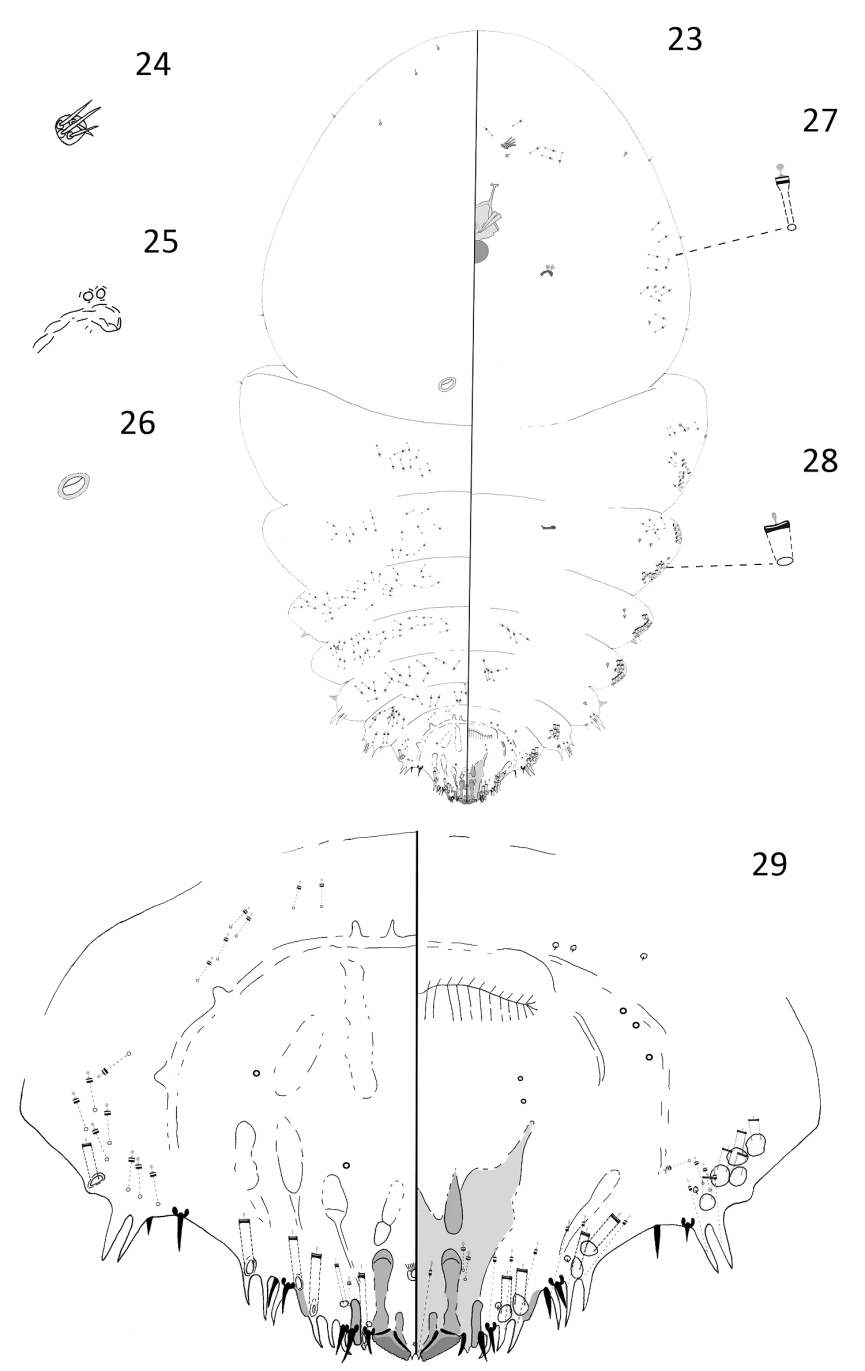
*Andaspis ornata* Hamilton and Williams, sp. n., adult female; **23** whole body **24** antenna **25** anterior spiracle **26** cicatrix **27** microduct **28** macroduct **29** pygidium.

#### Remarks.

The adult females of this species are different from all other species in the genus described so far, in having nine marginal macroducts located on the venter of the pygidium. This species is somewhat similar to *Andaspis retrusa* Williams, 1963, a species known to occur in India. Adult females of the two species have microducts present in groups across the body surface and have a second lobe. This species differs from *A. retrusa* by the following characters (those for *A. retrusa* in parentheses): five marginal macroducts on the dorsum (four marginal macroducts on the dorsum), one submarginal macroduct on the dorsum (numerous dorsal ducts on the dorsum), two scleroses arising from each median lobe (no scleroses arising from each median lobe), lacking perivulvar pores (five groups of perivulvar pores), and antennae with four setae (antennae with three setae).

#### Etymology.

The specific epithet *ornata* is the Latin feminine adjective meaning ornate and refers to the many marginal macroducts located on the pygidium.

### Key to adult females of *Andaspis* MacGillivray from New Caledonia

**Table:** 

1	Spurs present along anterior margin of head, body shape oblong	***Andaspis brevicornuta* sp. n.**
–	No spurs present along anterior margin of head; body shape fusiform, oblong, obovate, or oval	**2**
2	Cicatrix present on each side of mesothorax, anal opening located between scleroses on median lobes, and nine marginal macroducts located on venter	***Andaspis ornata* sp. n.**
–	Cicatrices absent on mesothorax, anal opening located near base (anterior edge) of pygidium, and two or fewer marginal macroducts located on venter	**3**
3	Five marginal macroducts located on the dorsum and two marginal macroducts located on the venter	***Andaspis novaecaledoniae* sp. n.**
–	Four marginal macroducts located on the dorsum and no marginal macroducts located on the venter	**4**
4	Body shape oval or oblong and with hourglass-shaped sclerosis located anterior to each second lobe	***Andaspis nothofagi* sp. n.**
–	Body shape fusiform and with a small pyriform sclerosis located anterior to each second lobe	***Andaspis conica* sp. n.**

## Supplementary Material

XML Treatment for Andaspis

XML Treatment for Andaspis brevicornuta

XML Treatment for Andaspis conica

XML Treatment for Andaspis nothofagi

XML Treatment for Andaspis novaecaledoniae

XML Treatment for Andaspis ornata
